# Perlecan/*Hspg2* Deficiency Alters the Pericellular Space of the Lacunocanalicular System Surrounding Osteocytic Processes in Cortical Bone

**DOI:** 10.1002/jbmr.236

**Published:** 2010-09-02

**Authors:** William R Thompson, Shannon Modla, Brian J Grindel, Kirk J Czymmek, Catherine B Kirn-Safran, Liyun Wang, Randall L Duncan, Mary C Farach-Carson

**Affiliations:** 1 Department of Physical Therapy, University of DelawareNewark, DE, USA; 2 Program in Biomechanics and Movement Science, University of DelawareNewark, DE, USA; 3 Delaware Biotechnology InstituteNewark, DE, USA; 4 Department of Biochemistry and Cell Biology, Rice UniversityHouston, TX, USA; 5 Department of Biological Sciences, University of DelawareNewark, DE, USA; 6 Department of Mechanical Engineering, University of DelawareNewark, DE, USA

**Keywords:** OSTEOCYTE, PERLECAN/HSPG2, MECHANOSENSING, HEPARAN SULFATE, LACUNOCANALICULAR SYSTEM, CORTICAL BONE

## Abstract

Osteocytes project long, slender processes throughout the mineralized matrix of bone, where they connect and communicate with effector cells. The interconnected cellular projections form the functional lacunocanalicular system, allowing fluid to pass for cell-to-cell communication and nutrient and waste exchange. Prevention of mineralization in the pericellular space of the lacunocanalicular pericellular space is crucial for uninhibited interstitial fluid movement. Factors contributing to the ability of the pericellular space of the lacunocanalicular system to remain open and unmineralized are unclear. Immunofluorescence and immunogold localization by transmission electron microscopy demonstrated perlecan/*Hspg2* signal localized to the osteocyte lacunocanalicular system of cortical bone, and this proteoglycan was found in the pericellular space of the lacunocanalicular system. In this study we examined osteocyte lacunocanalicular morphology in mice deficient in the large heparan sulfate proteoglycan perlecan/*Hspg2* in this tissue. Ultrastructural measurements with electron microscopy of perlecan/*Hspg2*-deficient mice demonstrated diminished osteocyte canalicular pericellular area, resulting from a reduction in the total canalicular area. Additionally, perlecan/Hspg2-deficient mice showed decreased canalicular density and a reduced number of transverse tethering elements per canaliculus. These data indicated that perlecan/*Hspg2* contributed to the integrity of the osteocyte lacunocanalicular system by maintaining the size of the pericellular space, an essential task to promote uninhibited interstitial fluid movement in this mechanosensitive environment. This work thus identified a new barrier function for perlecan/*Hspg2* in murine cortical bone. © 2011 American Society for Bone and Mineral Research.

## Introduction

Bone is a unique mineralized connective tissue that adapts its structure dynamically in response to its environment.(1) Osteocytes are the most abundant cell type of bone, accounting for approximately 90% of all bone cells,(2) and their primary function is to sense mechanical load.(3) These cells are embedded deep within the mineralized matrix and connect via long, slender dendrite-like processes encased in small channels of mineralized matrix called *canaliculi*.(4) Each canaliculus connects to the cavelike mineralized structure surrounding the osteocyte cell body, and together they make up the functional lacunocanalicular system (LCS).(4)

The pericellular space between the cell process of the osteocyte and the mineralized matrix is filled with interstitial fluid and a variety of extracellular matrix (ECM) molecules.(5) The exact role of this space and the fluid within it is incompletely understood; however, this fluid is thought to promote the transport of waste and nutrients to facilitate metabolism.(6) Additionally, fluid flow–induced shear stress initiates mechanosensory responses from bone cells.(5,7)

The dimensions of the LCS have been well characterized by electron microscopy(8); however, the ability of this system to maintain an open, unmineralized pericellular space while being deeply embedded in calcified tissue remains unclear. Inoue and colleagues have demonstrated that matrix metalloproteinases may help to prevent mineral deposition in the LCS.(9)

Fluid flow throughout the LCS induces shear stress that stimulates the dendrite-like processes within canaliculi. The mechanism of action behind this process is widely debated; however, You and colleagues proposed a strain amplification model to account for the ability of load-induced fluid shear stress to mechanically deform the osteocyte cell membrane on the order necessary to initiate a mechanosensitive response from osteocytes.(7) In this model, an uncharacterized organic matrix filled the pericellular space around the osteocyte processes. Transverse “tethering elements” were proposed to anchor the cell processes and center them within the canaliculi.(7)

The presence of transverse tethering elements in the LCS, a crucial component of the proposed model, was further validated by transmission electron microscopy (TEM) confirming the presence and quantity of the ultrastructural elements surrounding the osteocyte cell processes. High-resolution images showed transverse tethering elements connecting the osteocyte process to the mineralized matrix of bone across a pericellular space of approximately 78 nm.(8)

Proteoglycans represent a major component of the pericellular material surrounding osteocyte processes(10) and are thought to comprise the transverse tethering elements in the LCS.(8) The only heparan sulfate proteoglycan with sufficient size to span the pericellular space of the LCS is perlecan/*Hspg2* (PLN).(11,12) The name *perlecan* means “string of pearls,” given because of its appearance as globules separated by rods when imaged by TEM and atomic-force microscopy (AFM).(12,13) PLN is a very large five-domain heparan sulfate proteoglycan with a core protein of over 4000 amino acids.(14,15) The N-terminal domain I contains three GAG attachment sites, whereas the C-terminal domain V contains another variably used putative GAG attachment site.(16)

In addition to the size of PLN, various unique features of this molecule suggest several possible functions in the LCS of osteocytes. PLN is abundantly secreted into the pericellular space of numerous tissues, particularly near tissue barriers, and is ideally positioned to mediate signaling events by sequestering growth factors and binding integrins.(12,17–22) PLN and its long heparan sulfate chains regulate various physiologic functions in a variety of tissues where barriers are required, including separating epithelia and stroma, preventing cancer cell invasion,(23,24) maintaining the blood-brain barrier,(25) and controlling glomerular filtration and fluid movement.(26–29) Additionally, heparan sulfate inhibits hydroxyapatite (HAP) formation.(30,31)

In addition to recent evidence demonstrating the presence of tethering elements that span the pericellular space of the LCS, integrins, specifically β_3_-integrin, are expressed on the membranes of osteocyte processes.(32) These transmembrane proteins have been proposed to form focal attachments directly with regularly spaced protrusions or “hillocks” of the bone matrix wall within the osteocyte LCS.(32)

Proper maintenance of the pericellular space of the osteocyte LCS is essential for uninhibited interstitial fluid movement in cortical bone. The purpose of this study was to determine if the proteoglycan PLN is a component of the transverse tethering elements, discrete from the direct integrin-hillock links, in the pericellular space of the LCS of osteocytes. In this study we investigated the concept that PLN is positioned in the osteocyte LCS, where it functions to maintain the structural integrity of the open fluid-filled, unmineralized pericellular space. In vitro and in vivo cell systems and a PLN-deficient mouse model were used to examine this hypothesis.

## Materials and Methods

### Cell culture

Murine long bone osteocyte cells (MLO-Y4) were a generous gift from Dr Lynda Bonewald (University of Missouri–Kansas City, Kansas City, MO, USA). Cells were cultured in 100-mm tissue culture dishes (Corning, Inc., Corning, NY, USA) coated with rat tail type I collagen (0.15 mg/mL; BD Biosciences, San Jose, CA, USA) as described previously.(33) WiDr human colon carcinoma cells were cultured on T75 cell culture flasks (Fisher Scientific, Pittsburgh, PA, USA) as described previously.(34)

### mRNA isolation and PCR assays

MLO-Y4 cells were grown to 80% to 90% confluence (approximately 6 days in culture), and total RNA extracts were obtained using the RNeasy Kit (Qiagen, Valencia, CA, USA) with a typical yield of 400 to 800 ng/µL. mRNA extracts were treated with DNase using the DNA-*free* DNase Kit (Ambion, Austin, TX, USA) to remove DNA contamination. mRNA was reverse transcribed using the Omniscript Reverse Transcriptase Polymerase Chain Reaction (RT-PCR) Kit (Qiagen) according to the manufacturer's protocol. MLO-Y4 cDNA gene products then were amplified via conventional PCR using GoTaq Green PCR Master Mix (Promega, San Luis Obispo, CA, USA) and custom-designed primers against the murine PLN gene, *Hspg2*. Primers were designed using the PRIMER3 tool in the Biology Workbench online application (http://seqtool.sdsc.edu/CGI/BW.cgi, University of California, San Diego, CA, USA) from cDNA sequences published in the NCBI Nucleotide Database. Sequences were analyzed for optimal folding and hetero- and homodimerization patterns using the Integrated DNA Technologies OligoAnalyzer 3.1 (http://www.idtdna.com/analyzer/Applications/OligoAnalyzer/, Coralville, IA, USA) and for proper folding and dimerization. The NCBI Basic Local Alignment Search Tool (BLAST) also was used to ensure gene and species specificity (http://www.ncbi.nlm.nih.gov/BLAST). Primer sequences were as follows: 5'-CCCACTCTTGGACCCTGATA-3' and 5'-ATAGCTCCTCCTCTCTGGGC-3' for murine *Hspg2* (NCBI Accession No. M77174), generating a 94-bp product, and 5'-GATCATTGCTCCTCCTGAGC-3' and 5'-ACATCTGCTGGAAGGTGGAC-3' for murine *Actb* (NCBI Accession No. NM_007393), generating an 83-bp product. Gene products were visualized on an agarose gel (1.5%, w/v; Fisher Scientific) using ethidium bromide (4 × 10^−7^%, v/v; Fisher Scientific).

### Dot blot analysis

Conditioned medium from MLO-Y4 cells was collected after 6 days in culture at approximately 80% confluence. Conditioned medium from WiDr colon carcinoma cells that abundantly produce PLN (90% confluent) was collected and used as a positive control. Fresh medium was applied for both cell lines on day 3. Medium from each cell line (10 µL) was applied to the blotting apparatus (in triplicate) and allowed to bind to the nitrocellulose membrane by gravity flow at room temperature, as described previously.(35) Blots were blocked in Tris-buffered saline (TBS) containing Tween-20 (0.1% v/v; TBS-T) and bovine serum albumin (BSA, 3% w/v) for 4 hours at room temperature with rotary agitation. The anti-PLN mouse monoclonal Ab clone A76, as described previously,(36,37) was added to the blocking solution at a 1:500 dilution and incubated for 2 hours at room temperature with rotary agitation. The blot was rinsed three times with TBS-T and incubated with a goat anti-mouse–horseradish peroxidase (HRP) secondary Ab (1:100,000 dilution; Jackson Immunoresearch Laboratories, West Grove, PA, USA) in TBS-T. Following a 2-hour incubation with rotary agitation at room temperature and 3 × 5 minute washes with TBS-T, the secondary antibody was detected and visualized using the Super Signal West Dura substrate (Fisher Scientific) and brief film exposure.

### Mice

Mice deficient for PLN were generated in a C57BL/6J background as described previously and were a generous gift from Dr Kathryn Rodgers.(38) Briefly, retention of the neomycin selection cassette (C1532Yneo) on intron 16 located between exons encoding for PLN domain III-3 structure results in transcriptional defects leading to decreased PLN secretion that we describe as PLN deficiency in homozygous mutants. These mice exhibit dwarfism and skeletal defects, including pigeon breast, flat face, and hip dysplasia characteristic of the human condition Schwartz Jampel syndrome.(38) These mice have been referred to previously as *hypomorphic* owing to the retention of some wild-type (WT) function of the gene resulting in a less severe phenotype than a null allele.(39,40) WT animals and those heterozygous for the C1532Yneo mutation were reported to be undistinguishable phenotypically, and both were used as controls. All PLN-deficient mice or control mice were age-matched (8 to 9 month old) male littermates. All animal studies were performed with the approval of the University of Delaware institutional animal care and use committee (IACUC).

### Tissue preparation

To assess osteocyte ultrastructure in control and PLN-deficient mice, each femur was surgically dissected, cut into 1-mm segments with a diamond wafering saw, and immersion fixed with paraformaldehyde (4% v/v) and glutaraldehyde (2% v/v) with ruthenium hexamine trichloride (RHT, 0.7% w/v) in 0.05 M sodium cacodylate buffer as described previously.(8) Because of the potential interference by glutaraldehyde and RHT with antibody interactions, as well as to attain the best morphology possible in the absence of these additional fixatives, tissues used for immunogold assays were perfusion fixed with only paraformaldehyde (4% v/v) in 0.05 M sodium cacodylate buffer as described previously.(41) Briefly, mice were fixed by vascular perfusion using perfluorocompound FC-75 (13.3% v/v; Fisher Scientific), pluronic F-68 polyol (3.3% w/v; Fisher Scientific), paraformaldehyde (4% v/v; EMS, Hatfield, PA, USA), and sodium cacodylate buffer (0.05 M; EMS). Following administration of fixatives, the femurs and tibias were surgically removed, and all soft tissue was debrided from the bone. Each bone was cut into 1-mm cross sections using a diamond wafering saw, immediately immersed in fresh fixative containing paraformaldehyde (4% v/v) and calcium chloride (0.02 M) in sodium cacodylate buffer (0.05 M), and incubated at 4°C overnight. Bone sections were decalcified in EDTA (10% w/v) with paraformaldehyde (1% v/v) in Tris-HCl buffer (0.1 M, pH 7.4) as described previously.(8)

### Cell culture and cortical bone immunofluorescence

Approximately 1000 MLO-Y4 cells were seeded onto collagen I–coated 8-well chambers (NUNC, Rochester, NY, USA) and cultured as described previously.(33) When cells were 80% to 90% confluent, the medium was removed, cells were washed three times with TBS, and fixed with paraformaldehyde (4% v/v; EMS) diluted in TBS for 45 minutes at room temperature. Cells were washed three times with TBS to remove residual fixative and incubated for 1 hour in blocking solution consisting of BSA (3% w/v) and normal goat serum (10% v/v; Sigma, St Louis, MO, USA) diluted in TBS.

Decalcified bone sections were infiltrated with sucrose (2.3 M) in phosphate buffer (PB), immersed in tissue freezing medium (EMS), and stored at −80°C. Smaller sections (10 µm) were cut using a Leica CM3050 S cryostat microtome and transferred to adhesive glass slides using the Cryojane tape transfer system (Instrumedics, Inc., St Louis, MO, USA). Sections were washed with TBS to remove residual paraformaldehyde and blocked with BSA (3% w/v) and normal goat serum (2% v/v) in TBS.

Cells or bone sections were incubated with a mixture of four custom-designed anti-PLN rabbit polyclonal primary antibodies (Strategic Diagnostics, Inc., Newark, DE, USA) generated against human PLN domain IV sequences. Antibodies were diluted in blocking buffer (1:100), and cells or tissues were incubated with primary antibodies for 1 hour at room temperature. Following incubation with the primary antibodies, cells (4 × 10 minutes) or tissues (8 × 15 minutes) were washed with blocking solution and incubated with goat anti-rabbit Alexa Fluor 488 conjugated secondary Ab (1:200; Invitrogen, Carlsbad, CA, USA) and DRAQ5 nuclear stain (1:1000; Biostatus, Ltd., Shepshed Leicestershire, UK) diluted in blocking solution. Samples were washed 4 × 10 minutes with TBS, mounted, and stored at 4°C until imaged. Negative controls for cultured cells and bone sections were performed using nonimmune IgGs diluted at concentrations equivalent to primary antibodies or without primary antibodies. All samples were imaged with a Zeiss LSM 510 VIS confocal microscope using a 40× C-aprochromat water immersion objective (NA 1.2; Zeiss, Inc., Thornwood, NY, USA). Images were captured with an Axiovert 100M laser (Zeiss, Inc.) using the 488-, 543- and 633-nm laser lines for Alexa 488, Alexa 555, and Draq5, respectively.

### Ultrastructure imaging by TEM

Sections fixed with paraformaldehyde, glutaraldehyde, and RHT were dehydrated in an ascending series of ethanol (25%, 50%, 75%, and 95%) for 15 minutes each, left in ethanol (95%) overnight at 4°C, followed by 100% ethanol for 2 × 15 min. Quetol resin (EMS) was used to embed the sections according to the manufacturer's protocol. Briefly, a quetol/*n*-butyl glycidyl ether (NBGE) mixture was added to bone sections in ascending ratios (1:3, 1:1, 3:1, and 100%) for 2 hours each, followed by 100% quetol overnight and an additional 1 hour of incubation with 100% quetol. Resin then was allowed to polymerize at 60°C for 48 hours.

Bone sections were cut into ultrathin sections with a diamond knife, stained with a saturated solution of uranyl acetate in methanol, followed by Reynolds' lead citrate, and imaged with a Zeiss Libra 120 TEM at 120 kV. Images were acquired with a Gatan Ultrascan 1000 2k × 2k digital camera (Pleasanton, CA, USA).

### Immunogold

Immunogold labeling and imaging by TEM was used to determine the position and localization of PLN with optimal resolution within the LCS of cortical osteocytes. Tissue sectioning, blocking, and incubation with primary Abs recognizing epitopes primarily in domain IV of PLN were identical to the procedure described earlier for the immunofluorescence assays. Sections were labeled with goat anti-rabbit F(ab') ultrasmall gold conjugated secondary Ab (1:100; EMS) and silver enhanced according to the Aurion ultrasmall gold labeling protocol (Aurion, Wageningen, Netherlands). Negative controls were performed using nonimmune IgGs diluted at concentrations equivalent to primary antibodies or without primary antibodies. Sections were dehydrated, embedded in quetol resin, cut into ultrathin sections, stained with uranyl acetate and Reynolds' lead citrate, and viewed by TEM as described earlier.

### Analysis of canalicular ultrastructure

Canalicular pericellular area, total canalicular area, process area, canalicular density, and the number of tethering elements per canaliculus were analyzed from sections of control and PLN-deficient mouse femoral bone by TEM. Each femur was cut crosswise into 1-mm sections at midshaft and embedded in resin as described earlier for ultrastructural imaging. Sections used for TEM analysis were chosen randomly to provide a sampling along the femoral diaphysis. Multiple ultrathin sections were created from each 1-mm section, and images obtained from each ultrathin section were analyzed randomly so as not to create any analytical bias. Only cross-sectional images of canaliculi were included in the analysis. Pericellular area was determined using the ImageJ program (NIH, Bethesda, MD, USA). The total canalicular area and the area of each osteocyte process first were determined by outlining each structure using ImageJ, a method that reported the area of each outlined region. Next, the pericellular area was found by subtracting the process area from the total canalicular area. Canalicular density was assessed by determining the total number of cross-sectional canaliculi per total bone area. Longitudinal sections of canaliculi or canaliculi with poorly defined canalicular walls were omitted from all analyses to improve objectivity by including only cross-sectional canaliculi with well-defined canalicular walls. Tethering elements were counted for each canaliculus and for the purpose of this study were defined as projections emanating from the osteocyte process that span the pericellular space and are in contact with the canalicular wall, as illustrated previously.(8) Objects present in the pericellular space lacking these criteria were not included in the analysis. Ultrastructural analysis included images from three separate bone sections of each of three PLN-deficient mice and two control mice. Animal genotype was blinded during the analysis of all canalicular measurements. Significance was determined by two-tailed Student's *t* test and variance reported as SEM.

## Results

### Osteocyte ultrastructure

**Fig. 1:**
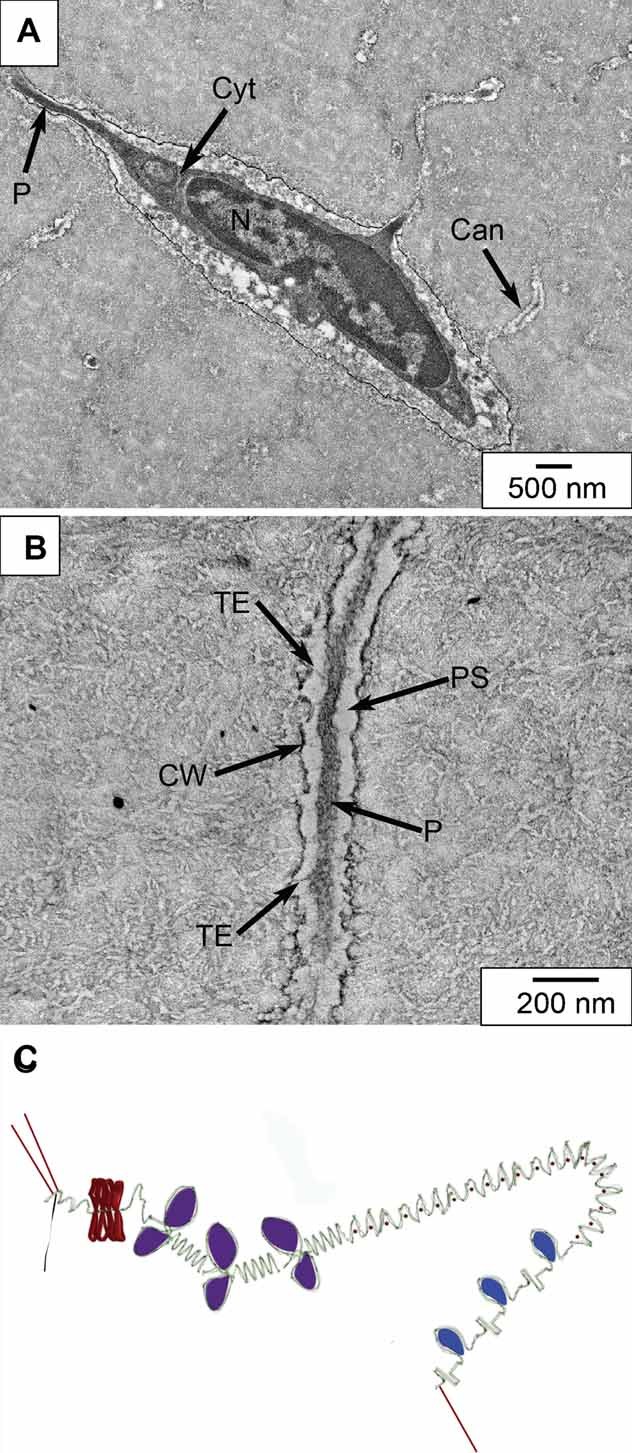
The molecular size of PLN is sufficient to span the pericellular space of the LCS. (*A*) Electron photomicrograph of a single cortical osteocyte with cytoplasmic processes (P), empty canaliculi (Can) extending from the cell body and with a prominent nucleus (N), and very little endoplasmic reticulum (ER) and Golgi-like structures in the cytosol (Cyt), features characteristic of osteocytes. (*B*) Electron micrograph of a cell process where the transverse tethering elements (TEs) clearly span the pericellular space (PS) between the process (P) and the canalicular wall (CW). (*C*) Schematic representation of PLN. The size of this large heparan sulfate proteoglycan is sufficient to span the pericellular space of the LCS of osteocytes.

TEM images of cortical mouse osteocytes were obtained to confirm the presence of the transverse tethering elements in the LCS and to determine the size relationship between the pericellular space and PLN. Figure 1*A* shows a single osteocyte with a prominent nucleus (N) and very little endoplasmic reticulum (ER) and Golgi-like structures in the cytosol (Cyt), features characteristic of osteocytes. Multiple canalicular projections (Can) protrude from the cell body, several of which are devoid of a process, or the plane of the section was in varying orientation, preventing full visualization of the process. A single canaliculus with a process (P) centered in it is seen emanating from the cell body (Fig. 1*A*). The ultrastructure of the osteocyte process is well defined in the enlarged image of the canaliculus (Fig. 1*B*). Distinct collagen bundles of the mineralized ECM are seen surrounding either side of the canaliculus. Transverse tethering elements (TEs) span the pericellular space (PS) between the process (P) and the canalicular wall (CW). The osteocyte process is centered in the canaliculus, and the pericellular space is seen distinctly. This space was previously measured to be approximately 78 nm, which is consistent with the data shown here (Fig. 1*B*). The cartoon image of PLN demonstrates the multidomain character of this large heparan sulfate proteoglycan (Fig. 1*C*). Notably, the four potential heparan sulfate chains, three on domain I and one on domain V, are shown. A compilation of studies indicates the approximate size of PLN to be 100 to 200 nm, making it the only heparan sulfate proteoglycan of which we are aware that could span the very large pericellular space of the osteocyte LCS.

### PLN localizes along the processes of MLO-Y4 cells and the LCS of osteocytes

**Fig. 2:**
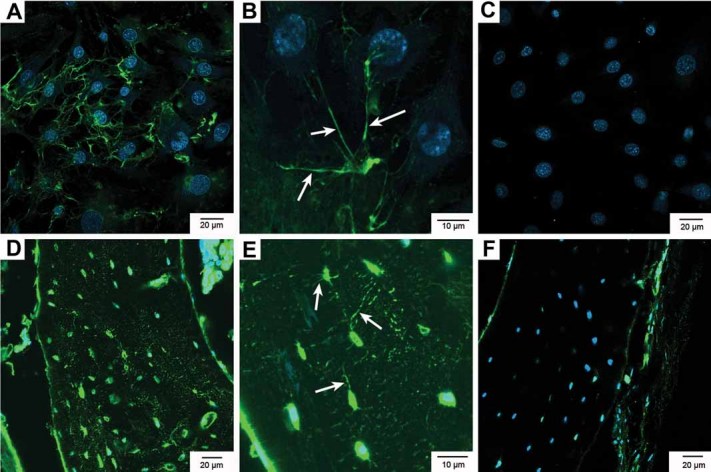
PLN localizes along the cytoplasmic processes of MLO-Y4 cells and the LCS of osteocytes in vivo. MLO-Y4 cells (*A*–*C*) were stained with either a mix of polyclonal PLN Abs (*A*, *B*) or with normal rabbit IgG Ab. MLO-Y4 cells display a unique expression pattern of PLN in a distribution resembling a network or matrix-like appearance (*A*) (representative of three separate experiments). PLN is prominently displayed along the cytoplasmic processes (*white arrows*) of MLO-Y4 cells (*B*) and absent in normal IgG controls (*C*). Cortical mouse osteocytes (*D–F*) express PLN abundantly (*D*) (representative of two experiments from two animals using three bone slices from each animal). Cell bodies and the LCS of osteocytes (*white arrows*) show robust PLN signal (*E*). No signal was observed in osteocytes treated with normal IgG as a control (*F*).

PLN displays a unique distribution on the membrane and dendritic processes of MLO-Y4 osteocyte-like cells (Fig. 2*A, B*) and in the LCS of cortical osteocytes in vivo (Fig. 2*D, E*). PLN is expressed in a unique pattern typical of extracellularly deposited matrix proteins on the surface of MLO-Y4 cells (Fig. 2*A*). To maintain the extracellular localization of labeling, no detergent or other permeabilizing reagents were added. The staining pattern demonstrated the ability of PLN to be distributed in a network-like fashion along and between the cytoplasmic projections of these osteocyte-like cells. This pattern is clearly delineated in the enlarged image in panel *B*, where PLN is seen localizing along the processes of several cells connected to each other (Fig. 2*B*). Normal rabbit IgG (Fig. 2*C*, *white arrows*) and omission of the primary Ab controls (data not shown) show an absence of signal, indicating the specificity of the fluorescent signal.

To demonstrate the ability of osteocytes to express PLN in vivo, mouse long bones were labeled with anti-PLN Abs (Fig. 2*D, E*). Strong fluorescent signal is observed along the cell bodies and in the LCS of cortical osteocytes (Fig. 2*D*). The enlarged image in panel *E* shows distinct labeling of numerous canaliculi; most notably, staining is observed along canaliculi that interconnect osteocytes (Fig. 2*E*, *white arrows*). There are various areas where small portions of canaliculi are stained, but the entire length of the process cannot be seen owing to the section and/or confocal plane orientation of single optical sections. Fluorescent labeling is absent around the cell bodies or within the canaliculi with normal rabbit IgG (Fig. 2*F*) or without primary antibody controls (data not shown).

### Expression of PLN in MLO-Y4 cells

**Fig. 3:**
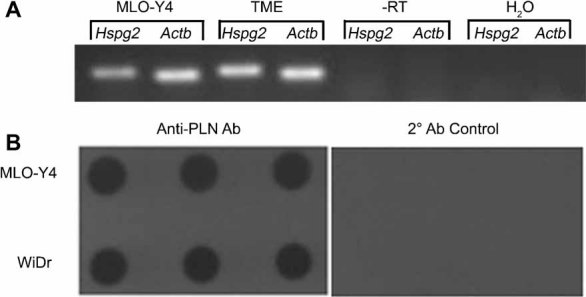
MLO-Y4 cells express *Hspg2* transcript and PLN protein. (*A*) *Hspg2* transcript expression was determined by RT-PCR. *Actb* transcript served as a load control and total mouse embryo (TME) RNA as a positive control. Omission of reverse transcriptase (–RT) and cDNA template (H_2_O) were used as negative controls (representative of three separate experiments). (*B*) Production and secretion of PLN protein in MLO-Y4 cells demonstrated by dot blot. Conditioned medium from WiDr cells was used as a positive control and omission of primary Ab as a negative control (performed in triplicate with three samples from the same culture dish).

MLO-Y4 osteocyte-like cells produce *Hspg2* transcript (Fig. 3*A*) and PLN protein (Fig. 3*B*). Amplification of *Hspg2* from MLO-Y4 cDNA demonstrates a strong band seen next to the *Actb* internal control. Amplification of *Hspg2* and *Actb* from total mouse embryo (TME) cDNA was used as a positive control to verify the specificity of the primers. Two negative controls were used, including the omission of reverse transcriptase (–RT) and the omission of cDNA template (H_2_O). No bands were observed for each of the negative control conditions. These results indicate that MLO-Y4 cells produce *Hspg2* mRNA.

A dot-blot was used to assess the production and secretion of PLN protein from MLO-Y4 cells (Fig. 3*B*). The dot-blot assay was chosen because of the large size and high extent of glycosylation of PLN. The core protein is about 460 kDa, and the multiple large heparan sulfate chains make this molecule even larger, thus highly diminishing its ability to move into a standard polyacrylamide gel to perform a Western blot. Conditioned medium from MLO-Y4 cells and WiDr human colon carcinoma cells, which express large amounts of PLN, were added in triplicate (three samples from the same plate) to each of two blots and probed with an anti-PLN Ab (mouse anti-PLN, clone A76) or with secondary Ab only as a negative control. Strong signal is observed for all three of the MLO-Y4 medium samples similar to that of WiDr positive control samples. The presence of PLN in conditioned medium validates the ability of MLO-Y4 cells to not only produce PLN but also to secrete this large heparan sulfate proteoglycan into the extracellular environment.

### PLN immunogold

**Fig. 4:**
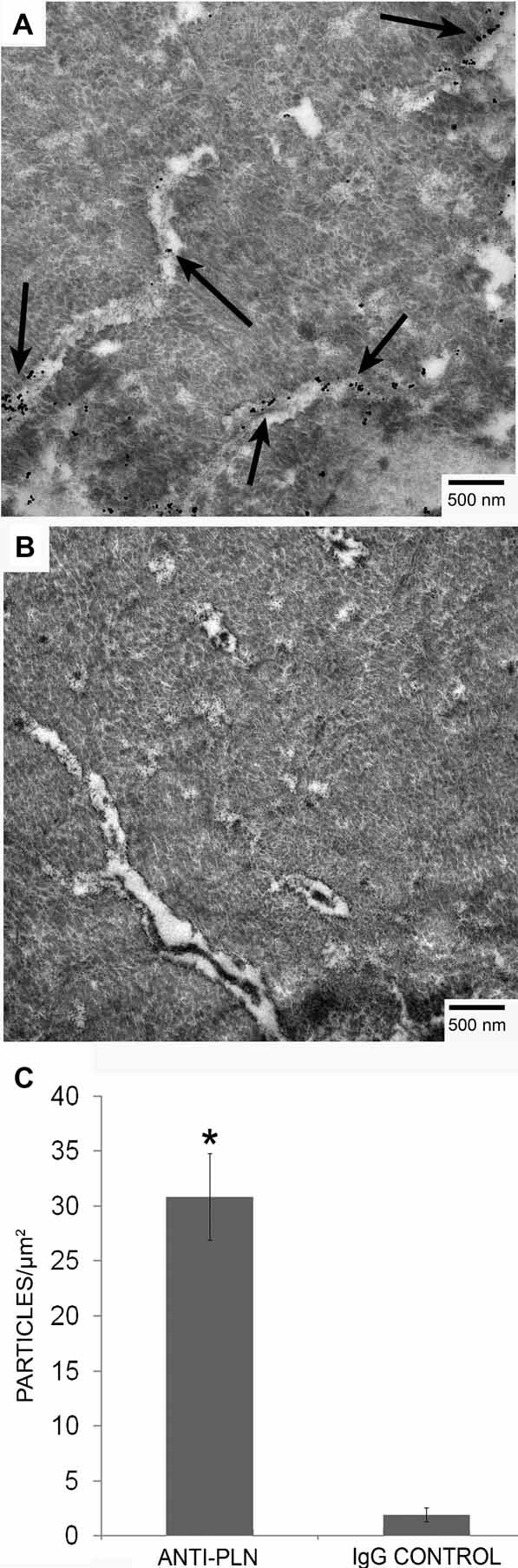
PLN resides within the pericellular space of the LCS. (*A*) Immunogold labeling of cortical bone demonstrates the presence of PLN within the pericellular space of osteocyte canaliculi, in the proper position to possibly serve as a component of the transverse tethering complex. Normal IgG labeling of the canaliculi (*B*) was significantly less than the PLN labeling (*A*, *black arrows*), as shown graphically in panel *C*. Data were compiled from a total area of 16 µm^2^ of canalicular area from approximately 50 canaliculi of anti-PLN- or normal IgG-treated bone (*p* < 0.001). Samples are representative of two experiments from random femoral bone sections of two animals. Statistical significance was determined using a two-tailed Student's *t* test, and variance is reported as SEM.

To assess the specific location of PLN within osteocyte canaliculi with a greater resolution than with confocal imaging, mouse bone was labeled with anti-PLN primary Ab followed by a gold-conjugated secondary Ab (Fig. 4*A*). Immunogold labeling was used to complement the immunofluorescence staining of cortical bone osteocytes to provide a more precise examination of the location of PLN relative to the pericellular space of the osteocyte LCS. Immunogold data provide enhanced evidence of PLN localization specifically within the pericellular space and not just within the vicinity of the canaliculi. PLN consistently labeled the pericellular space of osteocyte canaliculi and was seen in three separate canaliculi in Fig. 4*A* (*black arrows*). The differences in morphology between Figs. 1 and 4 are due to the mild fixatives necessary to preserve epitopes recognized by PLN Abs in Fig. 4. A clear pattern or periodicity of labeling along the canaliculi is not observed because the canaliculi project in and out of the plane of the section. PLN labeling was nearly always associated with the canaliculi (Fig. 4*A*, *arrows*) and not in a nonspecific fashion within the mineralized matrix. Very little to no labeling was observed along the canaliculi for the normal rabbit IgG control (Fig. 4*B*). The specificity of PLN labeling compared with the normal IgG control was assessed by analyzing approximately 50 canaliculi of each of three treated samples (for a total area of 16 µm^2^ of PLN and IgG-labeled bone). The ImageJ program was used to determine the area of each canaliculus, and the number of gold particles within this area was counted and averaged for all canaliculi assessed. The number of gold particles per square micron for the PLN-labeled bone was significantly greater than that of the normal rabbit IgG-labeled bone (*p* < .001; Fig. 4*C*).

### Canalicular structure in mice with PLN deficiency

**Fig. 5:**
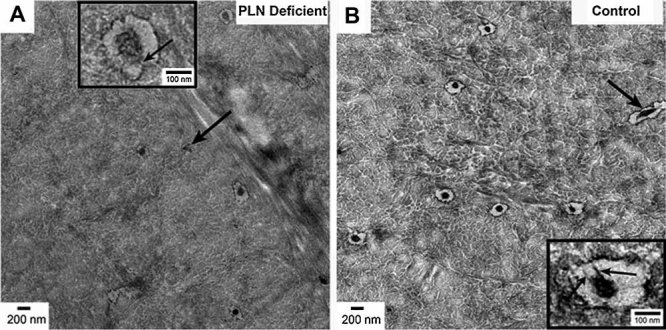
PLN-deficient mice have altered canalicular morphology. (*A*) PLN deficient mice demonstrate reduced pericellular area, decreased canalicular density, and fewer tethering elements per canaliculus than control mice (*B*). Insets show representative images of a single canaliculus to demonstrate the tethering elements (*arrows within insets*). The *black arrow* in panel *A* demonstrates a canaliculus that was omitted from analysis owing to an insufficiently defined canalicular wall. The *black arrow* in panel *B* indicates a canaliculus that was omitted from analysis because it appeared as a longitudinal section and not in cross section. Data are representative of measurements taken from multiple randomly selected ultrathin bone slices from three randomly selected 1-mm femoral bone sections taken from three PLN-deficient or two control mice.

**Fig. 6:**
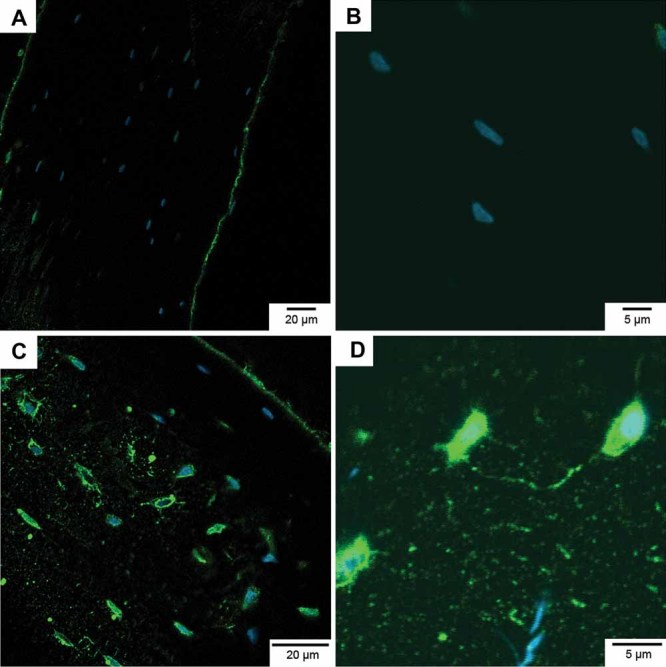
Cortical bone osteocytes of PLN-deficient mice express diminished levels of PLN. (*A*) Immunostaining for PLN in femurs of mice deficient for PLN showing decreased signal in cortical osteocytes. (*B*) High magnification of cortical osteocytes of PLN-deficient mice. (*C*) Immunostaining for PLN in control mouse cortical bone demonstrating strong signal around osteocyte cell bodies and canaliculi. (*D*) High magnification of cortical osteocytes in control mice showing strong immunofluorescent signal in osteocyte canaliculi that is absent in PLN-deficient osteocytes (*B*). Images are representative of two experiments from PLN-deficient or control mice using three bone sections from each animal.

PLN-deficient mice demonstrate significantly diminished pericellular area compared with control mice, constituting a decrease of 54.7% (*p* < .001; Table 1, Fig. 5). The total canalicular area is approximately 34% smaller in PLN-deficient mice than in control mice, which was a significant change (*p* < .001). In contrast, the process area tended to be slightly larger in PLN-deficient mice by 7.7%, but this difference was not significant (*p* > .05). PLN-deficient mice also showed a 23.4% decrease in canalicular density in cortical bone and a reduction in the number of tethering elements within each canaliculus by 35.8%, and both values were statistically significant (*p* < .01 and *p* < .001 respectively; Table 1). The appearance of the tethering elements is shown at high magnification in the insets to Fig. 5. Previous studies demonstrated approximately a 36% reduction in PLN transcript production in newborn endochondral skeleton by Northern blot analysis and only trace levels of PLN protein in long bones by immunostaining.(38) Here we report similar findings of severely diminished fluorescent signal of osteocytes from cortical bone sections labeled with anti-PLN antibodies (Fig. 6).

**Table 1:** Canalicular Ultrastructural Measurements^a^ of 8- to 9-Month-Old Male Mice

	Controls	PLN deficient	Percent change versus control
Pericellular area (µm^2^)	0.053 (± 0.002)	0.029 (± 0.001)	−54.7%***
Total canalicular area (µm^2^)	0.066 (± 0.002)	0.043 (± 0.001)	−34.8%***
Process area (µm^2^)	0.013 (± 0.0006)	0.014 (± 0.0005)	7.7%
Canalicular density (canaliculi/µm^2^)	0.124 (± 0.008)	0.095 (± 0.005)	−23.4%**
Tethering elements/canaliculus	3.223 (± 0.110)	2.069 (± 0.069)	−35.8%***

a Values reported represent the average measurements of 293 control canaliculi and 238 canaliculi from PLN-deficient mice. Total canalicular area includes both pericellular area plus process area.

* *p* < .01

** *p* < .001.

## Discussion

The cytoplasmic processes extending from osteocytes and the channels in which they reside have been implicated in several important physiologic functions, including mechanosensation, cell-cell communication, and waste/nutrient transport. Previous studies identify the presence of transverse elements spanning the pericellular space of the LCS(8); however, the molecular identity of these structures remains speculative. The results presented here identify the presence of the large heparan sulfate proteoglycan PLN well positioned within the osteocyte LCS to be part of an ECM complex in the pericellular space where structural integrity is essential for uninhibited fluid movement. Additionally, PLN deficiency resulted in significantly altered canalicular ultrastructural morphology, representing a novel phenotypic alteration involving this unique multifunctional ECM molecule.

Previous studies identified the presence of transverse tethering elements in the pericellular space and speculated that these elements were proteoglycans, glycosaminoglycans, or possibly wall protrusions linked to β_3_-integrins.(8,32) Although these studies did not take advantage of higher-resolution immunogold labeling to more precisely delineate integrin localization within the LCS, the distribution of staining correlated with protrusions, demonstrated using TEM, emanating from the canalicular wall, leading to the possibility that integrins may interact directly with the matrix wall by way of “hillocks” within the canalicular pericellular space. The hypothesis that integrins bind directly to the canalicular wall must be reconciled with previous observations showing an average osteocyte canalicular pericellular space of 78 nm.(8) In this study, we demonstrate distinct immunofluorescent labeling of PLN along numerous canaliculi in cortical bone. Higher-resolution immunologic labeling using immunogold with TEM imaging revealed that PLN antibodies were localized in the pericellular space of osteocyte canaliculi. Interestingly, the number of tethering elements in PLN-deficient cortical bone was significantly less than in controls. The localization of this molecule and the reduction of tethering elements in PLN-deficient mice strongly suggest that PLN is part of an ECM complex in the territorial matrix surrounding osteocytic processes. The tethering elements previously have been proposed to serve as a means of centering the dendritic processes with the canaliculi and potentially to function in mechanosensation.(7,8,32) While the tethering elements may consist of one or more molecular entities, the data presented here suggest that PLN is a component of the tethering complex. Additionally, PLN interacts with β_1_- and β_3_-integrins(18,21,42) and is the only heparan sulfate proteoglycan of sufficient size to span the pericellular space, leading to the possibility that an association between PLN and integrins may exist within the osteocyte LCS.

Previous studies demonstrated that osteocyte-like MLO-Y4 cells have a glycocalyx layer rich in hyaluronic acid but display only limited amounts of sulfated proteoglycans by alcian blue staining, thus contrasting with the data presented here showing distinct expression of the large heparan sulfate proteoglycan PLN in the osteocyte LCS.(43) Additional in vivo studies provide evidence for the expression of sulfated proteoglycans in the osteocyte LCS.(44–46) Specifically, chondroitin sulfate–rich proteoglycans discretely line the osteocyte lacunae and canaliculi, localizing along “fine filamentous structures” within the walls of the LCS in human alveolar bone.(44) Additionally, embryonic chick osteocytes express chondroitin sulfate, keratan sulfate, and dermatan sulfate along the walls of the lacunae and canaliculi.(45) The lack of sulfated proteoglycans described previously in MLO-Y4 cells may stem from differences in conditions related to cell culture versus in vivo bone samples because the dense extracellular environment of the in vivo LCS provides numerous potential binding partners for proteoglycans that may not be available when the mineralized matrix wall is absent. Moreover, Reilly and colleagues cultured MLO-Y4 cells for only 2 to 3 days, and it has been proposed that with additional time in culture, sulfated proteoglycans and glycosaminoglycans may become a component of the osteocyte glycocalyx.(43) While hyaluronic acid is a major component of the osteocyte glycocalyx and contributes to fluid flow–induced prostaglandin E_2_ (PGE_2_) release, it has not been implicated as a component of the tethering elements.(43) Sulfated proteoglycans are present alongside hyaluronic acid in the glycocalyx of endothelial cells,(47) providing the potential for interactions between these two entities within the LCS; however, the ability of PLN to interact with hyaluronic acid or hyaluronic acid–binding protein has not yet been established.

PLN-deficient mice have reduced pericellular canalicular area that can be attributed to diminished total canalicular area and is not due to enlargement of the cytoplasmic processes. One possible explanation to account for the smaller pericellular area is the reduced overall size of PLN-deficient mice. Reduced skeletal size of PLN-deficient mice may lead to proportionately decreased canaliculi; however, this does not account for the differences we observe in process area. PLN-deficient mice have osteocyte processes 7.7% larger than control animals, which was not statistically significant. If the observed differences in pericellular area were related to skeletal size, a proportionate decrease in the osteocyte process would be expected; however, this was not seen. In fact, the cytoplasmic processes were slightly larger than in control animals. Therefore, the data support the conclusion that the deficiency of PLN and its heparan sulfate chains likely allows encroachment of the mineralized canalicular wall on the osteocyte process. Complemented by the observation of a reduced number of tethering elements observed by TEM in PLN-deficient mice, these data suggest a role for PLN in maintenance of the structural integrity and morphology of the LCS.

Proteoglycans aid in the prevention of tissue mineralization,(48–50) and heparan sulfate is a potent inhibitor of hydroxyapatite (HAP) formation.(30) Although PLN is not ubiquitous, it is found in numerous tissues, most commonly at interfaces where a barrier is required. A lack of PLN is associated with barrier breakdown leading to pathologies such as cancer cell invasion,(23,24) disruption of the blood-brain barrier,(25) altered glomerular filtration,(26) and failure of the chondro-osseous junction in bone.(36,51) Brown and colleagues demonstrated a distinct downregulation of PLN at the chondro-osseous junction in murine hind limbs, with disappearance of PLN coinciding with the onset of mineralization.(36) Immunohistochemistry of murine hind limbs reveals a very strong PLN signal in chondrocytes and their matrix, yet virtually no PLN expression was observed in osteoblasts of trabecular bone or along the periosteal wall.(36) Additionally, Rodgers and colleagues noted increased hypertrophic calcified cartilage in the developing growth plates of PLN-deficient mice,(38) consistent with the proposed mineralization barrier function for PLN and heparan sulfate at the chondro-osseous junction.(36) The presence of PLN within the osteocyte LCS, as shown here and previous observations of sulfated proteoglycans localized to “fine filamentous material” in the walls of osteocyte canaliculi (but absent from the mineralized matrix of mature human alveolar bone)(44) support the hypothesis that while osteoblasts downregulate PLN expression to allow for proper matrix mineralization, osteocytes regain the ability to secrete PLN and other glycosaminoglycans. The presence of PLN within the LCS likely serves as a barrier for mineral formation, helping to maintain the size and structure of the pericellular space. Other potential functions include facilitating waste exchange, contributing to fluid flow–induced mechanosensation, and maintaining osteocyte viability.

This study demonstrated a reduction in the density of canaliculi in cortical bone of PLN-deficient mice, suggesting that PLN is able to regulate osteocyte LCS morphology. Previous studies demonstrated that normal bone is characterized by high osteocyte connectivity with processes oriented toward the vasculature.(52) In contrast, osteoporotic bone demonstrates decreased connectivity and a disorientation of the cellular processes. Osteoarthritic bone has diminished connectivity, but osteocyte process orientation is normal. Additionally, osteomalacic bone has a highly disorganized LCS with distorted processes.(52) Alterations of osteocyte canalicular density and morphologic arrangement are likely to have an effect on osteocyte function and possibly the mechanical properties of bone.(53) Potential mechanisms involving PLN in the establishment of normal bone mass will require further investigation.

In summary, our results identified PLN as a constituent of the LCS of murine cortical bone, where it plays a role in preserving the size of the pericellular space in the LCS, presumably by contributing to the structural integrity of the territorial matrix surrounding osteocytic processes. Previous studies identified the presence of transverse tethering elements in the pericellular space and speculated that these elements were proteoglycans, glycosaminoglycans, or possibly integrins.(8,32) Although the data presented here do not definitively identify PLN as the tethering element, our data indicate PLN to be well positioned in osteocyte canaliculi to contribute to the specialized matrix environment surrounding osteocyte processes and that PLN helps to maintain the unmineralized pericellular space of the LCS. In this study we showed that cortical bone with deficient PLN had fewer tethering elements per canaliculus and decreased density of canaliculi, leading to the possibility that heparan sulfate in the ECM in the pericellular space influences the formation and/or distribution of osteocyte processes. These data demonstrate a novel osteocyte phenotype involving the large heparan sulfate proteoglycan PLN, and further exploration of its role in maintenance of open canalicular structure and osteocyte function may reveal important molecular and functional insights into this specialized mechanoregulatory environment.
